# Reconsidering Hydrosols as Main Products of Aromatic Plants Manufactory: The Lavandin (*Lavandula* × *intermedia*) Case Study in Tuscany

**DOI:** 10.3390/molecules25092225

**Published:** 2020-05-09

**Authors:** Matteo Politi, Luigi Menghini, Barbara Conti, Stefano Bedini, Priscilla Farina, Pier Luigi Cioni, Alessandra Braca, Marinella De Leo

**Affiliations:** 1 Department of Pharmacy, University of Chieti-Pescara, via Vestini 1, 66100 Chieti Scalo, Italy; matteo.politi@unich.it (M.P.); luigi.menghini@unich.it (L.M.); 2 Department of Agriculture, Food and Environment, University of Pisa, via del Borghetto 80, 56124 Pisa, Italy; barbara.conti@unipi.it (B.C.); stefano.bedini@unipi.it (S.B.); priscilla.farina@phd.unipi.it (P.F.); 3 Department of Pharmacy, University of Pisa, via Bonanno 33, 56126 Pisa, Italy; pierluigi.cioni@libero.it (P.L.C.); marinella.deleo@unipi.it (M.D.L.); 4 CISUP, Centre for Instrumentation Sharing, Lungarno Pacinotti 43, 56126 Pisa, Italy

**Keywords:** *Lavandula* × *intermedia*, lavandin aromatic waters, hydrosol, hydrolate, fingerprinting analysis, insect repellence, germination inhibitors, multifunctional agriculture

## Abstract

The present work evaluates for the first time two Lavandin (*Lavandula × intermedia* Emeric ex Loisel.) aromatic waters obtained from different plant organs, the flowers and the stems. Both extracts were analysed by GC-MS, which indicates semi-quantitative differences between the major metabolites including linalool, 1,8-cineole, camphor, linalyl acetate and 4-terpineol. ^1^H-NMR and LC-MS investigation confirmed the presence of these compounds. Moreover, behavioural tests with the food insect pest *Tribolium confusum* (Coleoptera Tenebrionidae) showed a good repellency for both hydrosols extracts with RD_50_ values of 3.6 and 3.3 µL·cm^−2^ for the flowers and stems, respectively; at the higher concentrations, however, the hydrosol extract from the flowers is expected to be more effective than the one from the stems. The effect of the flowers and stems aromatic water of Lavandin on seed germination of *Raphanus sativus* was also evaluated. Results showed that seed germination was completely inhibited by flowers hydrolate, having a possible application as natural herbicide. The overall experience with these Lavandin extracts indicates the potential of improved hydrolates to become the main distillation products, rather than by-products, of the aromatic plants manufacturing; this stimulates further discussions about the potential positive impacts that such a shift could have in the context of ecopharmacognosy.

## 1. Introduction

Aromatic waters, sometimes referred to as hydrolates, hydrosols or floral waters, are the aqueous solutions that remain after steam-distilling or hydro-distilling botanical material [1]. Due to their biological and organoleptic properties, aromatic waters are used in the food, flavouring and cooking industry [2], cosmetic and perfumery industry [3], aromatherapy [4], along with agriculture as biological agents [5,6], insect pest repellents [7] and food sanitizers [8], between others. They are highly investigated for their antimicrobial and antioxidant potentials [9,10], as well as for their chemical characterization especially in comparison with the corresponding essential oils obtained from the same plant materials [11,12,13,14,15,16,17,18].

Although quality standards for this class of natural extracts are still missing in major pharmacopoeias [19], their global economic impact is projected to grow with a significant growth rate of 5.17% from 2019 to 2024 and reach a market value of USD 437 million by the end of 2024 [20]. Globally, hydrosols market in Europe is anticipated to be the dominating market with a market share of 39.91% in 2018. Italy is a hub of famous perfumes and fragrances and the increasing internal demand for organic ingredients in personal care is expected to influence the growth of the hydrosols market during the forecast period [20].

During the distillation process of any aromatic plant materials, the essential oils are usually considered the main products, while the plant material after the distillation (solid residues), as well as the aromatic waters, are classically considered by-products of this manufactory chain. The exploitation of such by-products can constitute a sustainable approach, according to the principles of the circular economy, to face the environmental issues derived by the generation of this huge amount of wastes. Therefore, scientists are attracted to investigate the potential high value compounds still retained in such kind of by-products, especially from plants belonging to the Lamiaceae family, and in particular *Lavandula* genus, such as Lavandin (*Lavandula × intermedia* Emeric ex Loisel.), whose agricultural production and essential oils industry is largely developed. The economic impact of Lavandin essential oils is globally recognized and tracked within the scientific literature since the middle of the past century [21] till recent [22]; the potential of Lavandin hydrolate, however, remains to be investigated in depth.

Previous studies on Lavandin by-products includes the solid residues from the industrial steam distillation that can constitute an available and affordable source of phenolic compounds, potentially useful as natural and safe antioxidants, among others, in foodstuffs [23]; particularly, in the case of Lavandin solid waste, coumarin and herniarin are the major detected compounds [24]. On the other hand, Lavandin hydrolate shows nematicidal potential [5] and has been tested directly on a Petit Verdot vineyards in order to determine if such treatment causes changes in wine aroma composition [25]. The latter experiment is an indication that aromatic waters can directly become a ready-to-use product, without considering them by-products anymore. The present research goes toward this direction, considering the potential of Lavandin hydrolate to become even the main product within a manufacturing chain of this aromatic plant. As detailed in the “Results and Discussion”, this could have positive impacts in relation with environmental issues and therefore ecopharmacognosy, defined as the study of sustainable, biologically active, natural resources [26].

Previous comparative phytochemical study between Lavandin essential oil and hydrosol [27] highlights specific qualitative and quantitative difference in terms of major aromatic detected compounds. In particular, 1,8-cineole, linalool oxide, camphor, linalool, geraniol and borneol appear to be relatively more abundant in the hydrosol while linalyl acetate, the major compound of the analyzed essential oil, was detected only in traces in the hydrosol [27].

The present work focuses for the first time on the phytochemical and biological investigation of Lavandin aromatic waters obtained from two different parts of the collected plant material, the stems and the flowers, respectively. In particular, the phytochemical analysis was performed using the HeadSpace, Solid Phase Micro Extraction coupled with Gas Chromatography Mass Spectrometry (HS-SPME-GC/MS) method [28], Nuclear Magnetic Resonance (NMR) spectroscopy, and Ultra High Performance Liquid Chromatography (UHPLC)-Diode Array Detection (DAD)-Electrospray Ionization (ESI)-High Resolution Mass Spectrometry (HR-MS). The biological assays consisted in testing the repellent activity against the food insect pest *Tribolium confusum* (Coleoptera Tenebrionidae) measured by the Area Preference Method [29], the allelopathic properties on the seeds of *Raphanus sativus*, as well as the stability by testing the presence of different microorganisms in the samples along a period of time. The aim was to evaluate the development of an improved Lavandin aromatic water by selecting specific plant parts for the distillation step. At the time of the study, a Lavandin hydrolate was regularly obtained as a by-product of the essential oil manufactory of the selected farm. Although no chemical or biological analysis was performed on this by-product extract, it represented a waste for the farm due to its poor organoleptic quality. This aspect helps to define the main scope of the present research. In fact, the aim was to exploit an improved, high quality hydrosol, showing first of all good organoleptic properties; this, in combination with potential biological effects, could allow the development of a Lavandin hydrosol as the main product, rather than a by-product, of the overall manufactory chain analysed in this work. The selected manufacturer, taken as a case study, was the biodynamic agro-farm “Le Tassinaie” located in Tuscany, Italy, where Lavandin is cultivated, collected and distilled.

## 2. Results

### 2.1. HS-SPME-GC/MS of Lavandin Aromatic Waters

Comparison between essential oil and aromatic water obtained from the distillation of the flowers (data not shown) indicated relevant differences concerning some major shared compounds such as linalool and 1,8-cineole; their relative amounts in the essential oil and aromatic water flower extracts were 52.6% versus 43.8% and 8.5% versus 25.4%, respectively. The first compound was therefore relatively more abundant in the oil while the latter in the water extract. Linalyl acetate, the third most abundant compound in the oil (7.8%), was present in low amount in the aromatic water (2.1%). These changes could be due to the different polarity of these compounds, therefore their solubility in lipophilic or hydrophilic solvents, as discussed in Section 3.1. Using the same distillation conditions, detailed in Materials and Methods, it was no possible to obtain the essential oil from the stems, therefore its comparison with the corresponding aromatic water was not acquired.

Comparison between the secondary metabolites detected by HS-SPME-GC/MS analysis in the aromatic waters obtained from the flowers and the stems are shown in Table 1. In total, 31 compounds were identified with percentage of 99.4% in flowers and 99.5% in stems, respectively. The identification of the compounds was obtained by the comparison of their retention times with those of pure authentic samples, comparing their linear retention indices (LRI) relative to a series of *n*-hydrocarbons, and on computer matching against commercial NIST 98 [30] and Adams [31], and also made possible by the use of a homemade library of mass spectra built up from pure substances and components of known essential oils, and MS literature data. The results refer only on semi-quantitative data, which means that they indicate the relative amount of a specific compound within the analysed sample, without any indication about its absolute quantification. In both samples the emission profile is mainly rich in oxygenated monoterpenes (96.1% in flowers versus 92.5% in stems) being linalool, 1,8-cineole and camphor, the major detected compounds. However, their relative amount changes within each specific hydrolate, being the first compound more abundant in aromatic water from the flowers, while the other two are slightly less abundant if compared with stem aromatic water. Monoterpene hydrocarbons are present in 2.2 and 2.5% in flowers and stems, respectively.

### 2.2. ^1^H-NMR and LC-MS Analyses of Lavandin Aromatic Waters

**Figure 1:**
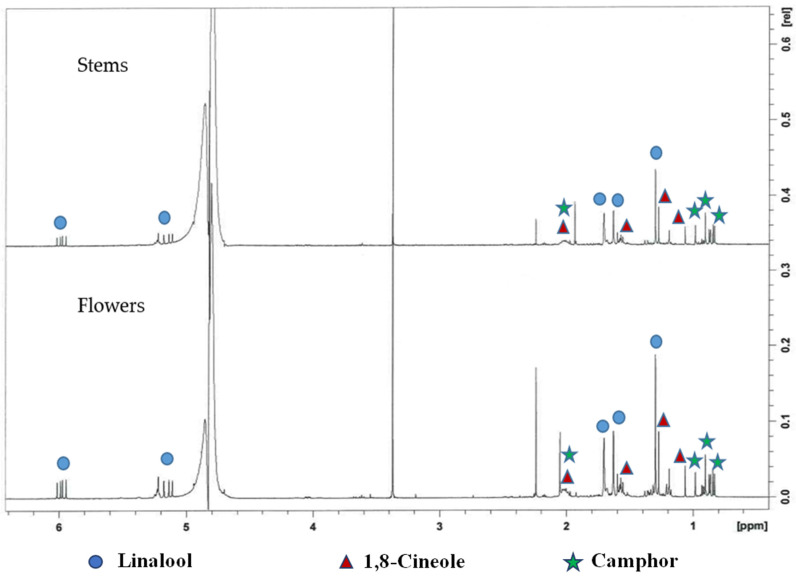
^1^H NMR spectra of Lavandin flowers and stems aromatic waters.

^1^H NMR with suppression of the water signal was here for the first time applied to directly analyse Lavandin aromatic waters. This technique, previously used to analyse different natural products water extracts as tinctures [32], allows the metabolite fingerprinting of the hydrolates without any manipulation of the sample other than the addition of 10% D_2_O for spectral acquisition. ^1^H NMR spectra of the two hydrolates are reported in Figure 1. The two spectra were almost superimposable and the signals could be attributable to linalool (δ 5.98, 5.23, 5.11, 1.70, 1.63 and 1.30), 1,8-cineole (δ 2.04, 1.63, 1.60, 1.28 and 1.08) and camphor (δ 2.21, 2.04, 1.51, 1.00, 0.92 and 0.85) molecules by comparison with literature data [33,34,35].

**Figure 2:**
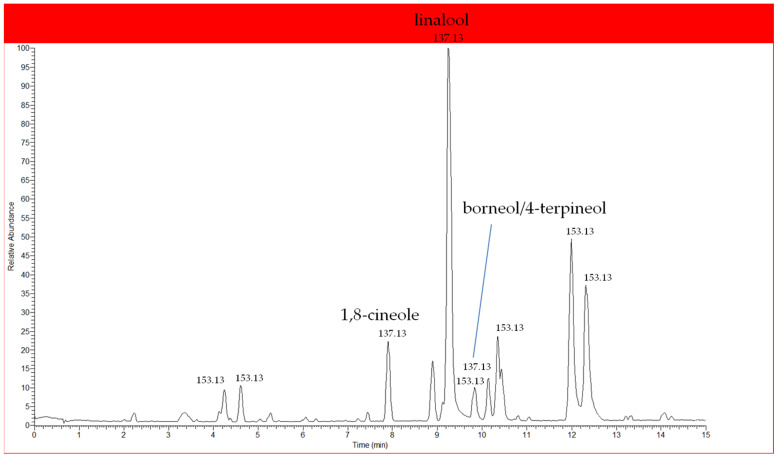
Ultra High Performance Liquid Chromatography (UHPLC)-MS (total ion current) chromatogram of Lavandin flower aromatic water, showing oxygenated monoterpenes, tentatively identified among the most abundant components as linaool, 1,8-cineole and borneol/ 4-terpineol.

Chemical analyses of Lavandin aromatic waters were also carried out by LC-DAD-HR-ESI-MS/MS technique. This approach was used to investigate if hydrolates were composed by interesting not volatile constituents that cannot be investigated by GC-MS. Few studies are reported in the literature investigating hydrolates by HPLC and they are focused on quantification of selected volatile analytes by DAD [36,37,38]. Results did not show the presence of characteristic constituents of Lavandin, such as phenols that Torras-Claveria et al. [39] identified in Lavandin waste after distillation of the essential oil, but only several monoterpenes detected already by GC-MS analysis. These findings emerged from LC-MS analyses in both positive and negative ion modes, suggesting that phenols can be found in Lavandin waste obtained after the distillation of essential oil, as previously demonstrated [36], but not in aromatic water that on the contrary is enriched only by volatile substances. Detection of volatile molecules is limited by the ESI source, more suitable for polar compounds. Analyses of full MS spectra and product ions generated during MS/MS experiments led to the tentative identification of the major peaks. According to HS-SPME-GC/MS analyses, flowers aromatic water showed four major peaks that can be attributed to oxygenated monoterpenes (Figure 2). All peaks showed the same molecular parent ion at *m*/*z* 137.13 and in all cases MS/MS experiments generated principal product ions at *m*/*z* 96 and 81. Thus, the four peaks could be attributed to: a) precursor ions [M+H-H_2_O]^+^ at *m*/*z* 137.13 of the oxygenated monoterpenes linalool (M = 154), 1,8-cineole (M = 154), borneol (M = 154), 4-terpineol (M = 154), generated in the ESI source by the loss of a water molecule [40]; b) precursor ion [M+H-CH_3_COOH]^+^ at *m*/*z* 137.13 of the oxygenated monoterpene linalyl acetate (M = 196) and/or lavandulyl acetate (M = 196) due to the loss of an acetic acid molecule in the ESI source [40]; d) less likely protonated molecular ion [M + H]^+^ at *m*/*z* 137.13 of monoterpene hydrocarbon myrcene (M = 136). Since acetyl derivatives have low solubility in water, these peaks could be attributed to the monoterpene alcohols linalool, 1,8-cineole, borneol and/or 4-terpineol. Other peaks showed precursor ions at *m*/*z* 153.13 characterized by the same fragmentation pattern (*m*/*z* at 135, 107, 93, 81 and 71), that were not identified since very limited data are reported in the literature about volatile compounds analysed by using ESI-LC-MS analyses and, to our knowledge, no previous analyses were performed on aromatic water using this technique. Based on MS data, one of these peaks could be assigned to the protonated molecular ion [M + H]^+^ of camphor (M = 152) that represents one of the main components detected in hydrolates.

### 2.3. Insect Repellence Bioassay

**Figure 3:**
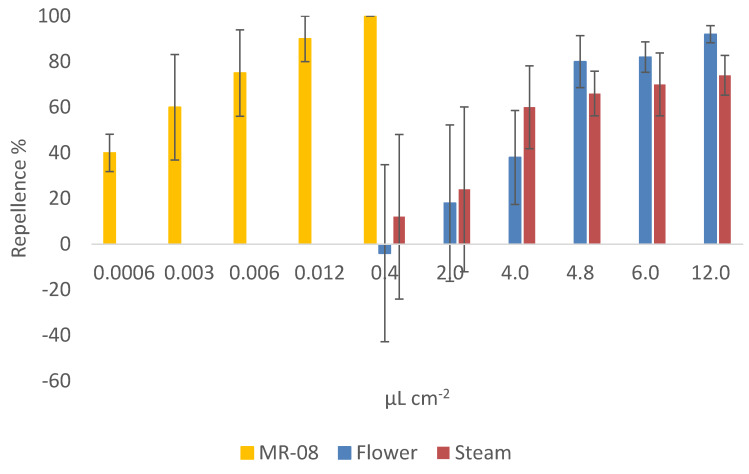
Mean repellence (%) of Lavandin flowers and stems aromatic waters and of the synthetic repellent MR-08 against *Tribolium confusum* adults. Bars represent standard error.

The repellence bioassay showed a clear effect of the extracts of Lavandin on the behaviour of *T. confusum* adults (Figure 3).

The repellent effect of the two Lavandin extracts was, however, lower than the one of the synthetic repellent MR-08 (ANCOVA *F*_2, 72_ = 4.010; *p* = 0.022; *η_p_^2^* = 0.10). In particular, the *post-hoc* tests of the repellence estimated marginal (EM) means (Table 2) indicated a significant difference between flowers extract and MR-08 (Bonferroni pairwise comparison, *p* = 0.042) and between stems extract and MR-08 (Bonferroni pairwise comparison, *p* = 0.024), but no differences between flowers and stems hydrosols (Bonferroni pairwise comparison, *p* = 1.000).

**Figure 4:**
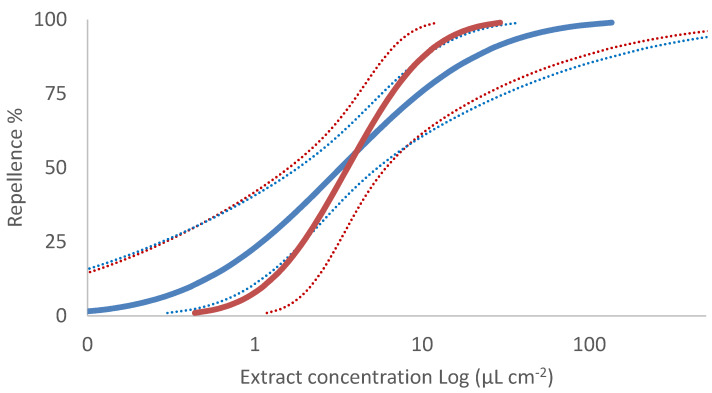
Probit model of the repellent effect (Repellence%) of Lavandin flowers (red line) and stems (blue line) hydrosols against *Tribolium confusum* adults. Dotted lines indicate 95% confidence intervals.

In line with the ANCOVA, the probit model showed similar RD_50_ values for both the Lavandin extracts (RD_50_ = 3.58 and 3.26 μL·cm^−2^ for flowers and stems, respectively) (Table 3). The different slopes of the probit regressions, however, indicate that, at the increase of the concentration, the activity of the flowers extract increases more rapidly than the stems one. Hence, at the higher concentrations the flowers extract is expected to be more effective than the stems one (RD_99_ = 29.30 and 136.07 μL·cm^−2^ for flowers and stems extracts, respectively) (Table 3) even if, the overlapping of the confidence intervals indicates that the difference is not significant (Figure 4).

### 2.4. Allelopathic Activity

**Figure 5:**
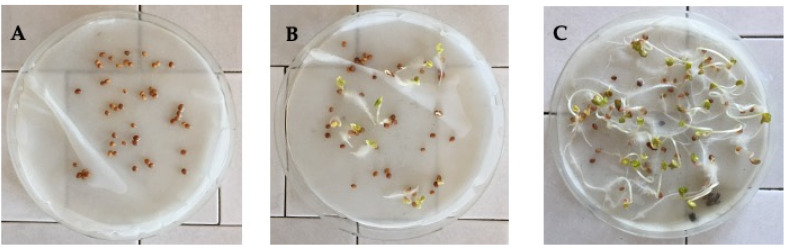
Inhibition of *Raphanus sativus* seed germination by Lavandin flowers and stems aromatic waters. (**A**) *Raphanus sativus* seed + flowers aromatic water; (**B**) *Raphanus sativus* seed + stems aromatic water; (**C**) *Raphanus sativus* seed + H_2_O = control.

The effect of the flowers and stems aromatic water of Lavandin on seed germination of tested species *Raphanus sativus* was evaluated (Table 4, Figure 5). Results showed that seed germination was completely inhibited by flowers hydrolate, while the stem aromatic water reduced the germination percentage to 24%.

### 2.5. Stability Assay

This experiment was performed only on the aromatic water obtained from the flowers, considered the best candidate for a possible commercial development; all the tests performed (see Section 4.6 in Materials and Methods) gave negative results and no colony growth was observed at least until month 12 (data not shown). As far as we know, this is the first time that a stability assay on a sample of aromatic water is reported within the scientific literature.

## 3. Discussion

### 3.1. Phytochemical Analysis

Linalool, which has good solubility in water (1.6 g L^−1^), is the most abundant compound detected by GC-MS in both the aromatic waters and the essential oil flower extracts (these latter data not shown) here analysed. Linalyl acetate is usually one of the most abundant detected volatiles in Lavandin essential oil [27,41], although its detection can vary significantly depending on the method adopted, direct GC/MS analysis or HS-SPME-GC/MS [41]. Linalyl acetate has a very low solubility in water (8.2 mg/L) and this could explain its low concentration in the Lavandin aromatic water here analysed. On the other hand, the major amount of 1,8-cineole observed in the aromatic water compared with the essential oil is likely to be due to the good solubility of this compound in water (3.5 g L^−1^). Chemical analyses performed by ^1^H-NMR and LC-MS techniques did not evidence the presence of not volatiles components but confirmed linalool and 1,8-cineole as the most represented ones.

Linalool is an important chemical fragrance, frequently used in scented products because of its fresh, flowery odour [42]. On the other hand, 1,8-cineole is classified as insect repellents defined as a substance causing insects to turn away from it or reject it as food [43]. The relative amount of both compounds suggested a possible better combination between a pleasant scent and a regular insect repellent activity for the extract obtained from the flowers compared to the aromatic water from the stems. In the latter case, the lower amount of linalool combined also with a higher amount of camphor, that is known to have a mothball-like odour [44], could be responsible for the unpleasant scent associated with this extract; this was also anecdotally reported by a group of non-trained informants selected within “Le Tassinaie” employees and customers during the period of the study.

### 3.2. Insect Repellence Bioassay

In the repellence experiment, it was observed for the first time a clear repellence of the hydrosols against the main food insect pest *T. confusum*. Although the repellent effect of aromatic plant essential oils has been extensively evaluated for their insecticidal and repellent activity, very few information is available on the effect of the hydrosols on insect pests and, to our knowledge, no information at all is available on their effect on food insect pest or Coleoptera in general. However, in line with our findings, a bioactivity of hydrosols has been observed against problematic mosquitoes, aphids, mites and nematods. Rabha and colleagues [45] observed a larvicidal activity of the hydrolates extracted from *Zanthoxylum limonella* (Dennst.) Alston (Rutaceae), *Zingiber officinale* Roscoe (Zingiberaceae)*, Curcuma longa* L. (Zingiberaceae) and *Cymbopogon citratus* (DC.) Stapf (Poaceae) against *Aedes albopictus* and *Culex quinquefasciatus* Say (Diptera: Culicidae) with LC_50_ values ranging from 11 to 40% *v*/*v*. On the contrary, the hydrolates of *Rosmarinus officinalis* L. (Lamiaceae) showed no repellent, irritant or toxic effects on the mosquito *Anopheles gambiae* Giles [46]. As for the aphids and mites, the hydrosols derived from *Ocimum basilicum* L. (Lamiaceae) and *Ruta chalepensis* L. (Rutaceae) caused significant mortality rates (between 46 and 64%) and a significant reduction of the fecundity of the cotton aphid *Aphis gossypii* Glover (Hemiptera: Aphididae) and of the two-spotted spider mite *Tetranychus urticae* Koch (Acari: Tetranychidae) major pests of cucumber and other crops [47]. A good toxic activity against mites was also observed by Petrakis and colleagues [48] for the hydrolates extracted from of *Origanum majorana* L., *Mentha pulegium* L. and *Melissa officinalis* L. (Lamiaceae). Recently, Sainz and colleagues [49] showed that the hydrolate of the rare plant *Artemisia pedemontana* subsp. *Assoana* (Willk.) Rivas Mart. (Asteraceae) is toxic to the plant-pathogenic nematode *Meloidogyne javanica*. Similar nematicidal effects have been also reported for the hydrolates from *Artemisia absinthium* L. (Asteraceae) [50], *Lavandula* × *intermedia* Emeric ex Loisel., *Lavandula stoechas* subsp. *luisieri* (Rozeira) Rozeira, *Thymus vulgaris* L. and *T. zygis* L. [5,6].

### 3.3. Herbicide Potential

One of the challenges of modern agriculture, which has the responsibility to feed a rapidly growing population, is to minimize the yield loss due also to the presence of undesired weeds [51]. In this context, further efforts are necessary for the development of environmental-friendly alternative to synthetic herbicides, being the latter often a matter of concern especially in terms of sustainability [52]. Natural products are therefore particularly attractive compounds that could inspire a new generation of eco-friendly herbicides [53]. The preliminary results acquired on the aromatic waters analysed in this work suggested the potential application of this class of derivatives as natural herbicides. In particular, the aromatic water obtained from the flowers, compared to that one from the stems, showed a greater potential in this regard. Plants belonging to the Lamiaceae family are reported as weed germination inhibitors due to the phytotoxicity of monoterpenes [54] among which 1,8-cineole and camphor, two of the main components of Lavandin hydrosols here analysed. Furthermore, open-chain and monocyclic alcohols were shown to be more active than hydrocarbons [55]. These evidences could explain why aromatic water from Lavandin flowers, containing higher content of linalool and 4-terpineol, showed stronger inhibition effect compared to aromatic water from stems.

### 3.4. Stability Assay

Concerning the stability assay, it is worthy to mention that hydrosols, being water base extracts, are potentially exposed to possible deterioration due to the growth of different kind of microorganisms, especially if compared with essential oils. In the current regulatory framework with a lack of defined standards for this class of natural extracts [19], it is not clear if the use of stabilizers should be mandatory, recommended or even unnecessary. Aromatic waters are the results of a distillation process and this should guarantee the absence of microorganism including spores in these classes of extracts. Moreover, the presence of aromatic derivatives having in many cases antimicrobial effects could be considered an additional advantage that could assure about the stability of the aromatic waters without the need of any sort of added stabilizer. For the case study of Lavandin hydrosol here analysed, the result of the stability assay clearly indicates that the use of additional stabilizers is not necessary; at least for the period of 12 months, during which several microbiological assays have been performed, no microorganism was detected within the extract. In any case, if the choice goes toward the development of aromatic waters without the use of external stabilizers, our recommendation is to spend particular attention to avoid cross-contaminations during the bottling or any other potentially critical step.

### 3.5. Ethnobotany and Ecopharmacognosy Considerations

The term “ethnobotany” was first used during a lecture in 1895 and defined as the study of “plants produced by primitive and indigenous peoples” [56]. Perhaps due to this initial definition, this term still can remind to something exotic and belonging to the past, which is basically wrong. After that, in fact, many researchers have defined ethnobotany in different contexts, and it is now generally accepted as the study of the relationship between plants and humans in their culture, where the term “ethno” refers to the study of people and “botany” to the study of plants [57]. This is a multidisciplinary area embracing the study archaeology, chemistry, botany, ecology, anthropology, linguistics, history, pharmacology, medicine, pharmacy, sociology, religion and mythology between others. An in-depth ethnobotanical research is therefore not so easy or straightforward and should require collaborations between different areas of expertise; it is difficult, in fact, to imagine a modern scientist to hold a consistent background in such diverse disciplines. As discussed later especially in Section 3.4, the result is that, quite often, ethnobotanical researches are rigorous in some aspects, while remaining superficial in others. Hopefully, a new generation of researchers specifically trained in ethnobotany will be able to overcome such limitations.

The recent term ‘‘ecopharmacognosy’’ refers to the study of “sustainable, biologically active, natural resources”. It is also considered a philosophical approach and a conceptual framework that may improve the access to food and health care products, assuring at the same time beneficial outcomes. Ecopharmacognosy introduces a new paradigm in natural product research and it challenges to think with respect to the environment [27]. In our understanding, this term allows the introduction of broad concepts such as sustainability, climate change, fair trade and planetary health between others, within the classical research approach in phytochemistry and pharmacognosy. We take this opportunity to discuss in the following sections, some aspects of this new philosophical shift in natural product research.

#### 3.5.1. Agricultural Methods and Climate Change

The productive capacity of land resources is considered a matter of concern in relation with the ecological sustainability in the context of modern agriculture [58]. Contemporary agricultural practices, in fact, are characterized by high rates of mechanization and the use of synthetic chemicals. Both factors contribute to soil erosion and nutrient loss [59]. This process has substantially contributed to climate change due to resulting greenhouse gases emissions [60], which is often combined with deforestation. The emergence of sustainability induces approaches such as the regenerative farming or others environmentally benign agricultural practices [61].

Despite an initial scepticism about biodynamic agriculture within the scientific community, a fair share of the available peer-reviewed research results of controlled field experiments as well as case studies show positive effects of the biodynamic method on yield, soil quality, biodiversity and environmental impact in terms of energy use and efficiency [62]. This kind of agricultural approach is generally practiced at lower scale compared with the industrialized methods, and usually includes low rates of mechanization and chemical input; all aspects that were observed also within the agro-farm that purchased the samples for the present work. Biodynamic is therefore considered one of the sustainable agriculture methods showing potential for mitigating some detrimental effects of chemical-dependent conventional agriculture [63]. Considering that most of the companies involved in the global trade and production of herbal remedies and other botanical products use plant material derived from cultivated sources rather than wild harvested (although, considering the species numbers rather than volume of material, the figures are generally inverted) [64], the choice of agricultural methods become particularly relevant within the context of ecopharmacognosy, which claims to pay much attention on climate change issues [65].

As a basic strategy, organic farming, or even more regenerative agricultural strategies such as the biodynamic one here discussed, should be encouraged through scientific opinions and evidence-based data. To address the increasing number of population and therefore goods demands including those related with herbal products, increasing the number of small-scale, sustainable and regenerative farms could offer a more ecologic alternative compared to that mainstream, large-scale, conventional agro-farm industries. This could trigger several snowball effects that are directly linked with climate change. The reduction of mechanization and chemical use are generally linked with an increasing biodiversity within the farm, as observed for the case described in this work. Low-tech agricultural implies that most of the tasks in the field are manually performed, and this increases the number of working opportunities in the agricultural contexts. The health benefit of horticultural and gardening activities are largely recognized [66] and this indicates that low-tech agricultural approach could be beneficial not only for the environment but also for humans; this perfectly matches the objectives of planetary health projects that are developed to address the health of human civilization and the state of the natural systems [67,68]; these are therefore relevant aspects to be considered within the context of ecopharmacognosy.

#### 3.5.2. Traditional Medicine and Paradigm Shift

Ecopharmacognosy is also deeply involved in the discussion about traditional plants medicine worldwide [69], claiming that modern scientific approaches and techniques must enhance the quality, assure the safety and demonstrate the efficacy of traditional herbal products. However, as Etkin suggests [70], when addressing concepts of efficacy in herbal medicine, certain practices may have no verifiable molecular reality. In fact, healing properties are often tightly woven into a broader and complex health cosmology that goes beyond molecular science. Hsu [71] refers to spells and incantations, among others, as important techniques to consider in order to understand the overall effectiveness of such kind of preparation. As stated by Reyes Garcia, “the focus on testing the active compounds of indigenous pharmacopoeias conveys the idea that local medicines become meaningful only when pharmacologically validated, and thus diminishes traditional knowledge systems and indigenous explanations of the world” [72]. This means that, once approaching traditional medicines, a paradigm shift is often required to properly understand the subject of study. Due to the lack of preparation especially of chemists, pharmacists and pharmacologists on ethno-related sciences, close collaborations with experts in these fields, usually anthropologists, are strongly recommendable.

Recently, an ethnobotanical and ethnopharmaceutical survey in the Amazonian jungle [73] indicates the relevance of giving recognition to the traditional methods of preparation of herbal remedies, challenging the actual importance of testing the quality, safety and efficacy of traditional medicines by measuring only the currently known scientific chemical and biological parameters. In the cited work [73], the phenomenon of human-plant interaction, deeply described within the current ethnobotanical and anthropological literature [74,75,76], represents the most relevant local parameter used to evaluate the concept of quality, safety and efficacy from the traditional perspective. The “good practices” applied in modern pharmaceutical science represent a very different set of standards than those applied in traditional medicine. Researches on traditional good practices are largely missing within in the ethnobotany and ethnopharmacology literature, while they could be a source of beneficial insights toward eco-friendly attitudes. This is true especially considering that the history of forest and agricultural landscape management practices of indigenous and local communities based on their traditional knowledge offer insights into principles and approaches that may be effective in coping with, and adapting to, climate change in the years ahead [77]. The concept of human-plants interaction observed in the Peruvian traditional medicine [73] resonates in a way with the typical small-scale farm agricultural activities, as for the case observed in the present work in relation with the Lavandin preparation, where most of the tasks are performed manually; this working attitude put more in a direct contact humans and plants. Human–plant interaction can be limited or even lost by practicing mechanical farming and this could be one reason more for having negative impacts on human and planetary health if using hi-tech agricultural approaches. Therefore, the pursuit of the mere scientific reasoning to measure traditional medicines without even considering the traditional perspectives on how to obtain high quality, as well as safe and effective drugs, could inhibit new understandings on possible sustainable managing of natural resources.

## 4. Materials and Methods

### 4.1. Plant Cultivation and Samples Preparation

The plants of Lavandin (*Lavandula × intermedia* Emeric ex Loisel.) were grown following the biodynamic agricultural principles [62], in the agro-farm “Le Tassinaie”, consisting in a main house surrounded by 3 hectares of land mostly dedicated to agricultural activities, located in the territory of Castellina Marittima, within the district of Pisa, in Tuscany region, Italy. The flowering aerial parts of Lavandin were manually collected in June 2017; the separation of the flowers from the stems, also performed manually, was achieved during the same day. The flowers yield was around 1/3 in weight of the overall collected plant material; the remaining 2/3 was made of stems and small leaves. Both parts of the plant materials were distilled separately during the same day using a TredTechnology extractor of essential oils, model EOE20. In both cases, 400 g of plant material were immersed in 3 L of tap water and distilled at the atmospheric pressure for 1 h, obtaining in both cases around 1 L of aromatic water. For the distillation of the stems, no significant yield of essential oil was observed, while for the distillation of the flowers, around 13 mL of essential oil was also obtained; this was separated by removing it from the top of the aromatic water with a glass syringe.

### 4.2. HS-SPME-GC/MS Analysis

The analysis of volatile compounds was performed by the Solid-Phase Micro-Extraction (SPME) technique, using a Supelco SPME devices coated with polydimethylsiloxane (PDMS, 100 μm). 2 mL of flowers and stems aromatic waters were put into a 5 mL flask and allowed to equilibrate for 30 min at room temperature. The fibre, previously conditioned according to the manufacturer recommendations, was then exposed to the headspace of each sample for 1 sec. Sampling was performed in an air-conditioned room (22 ± 1 °C) to guarantee a stable temperature during sampling. After the sampling time, the fibre was withdrawn into the needle, then transferred immediately to the injection port of the GC/MS, where the fibre was desorbed with a splitless injection method. Gas chromatography-electron impact mass spectrometry (GC/EI-MS) analyses were performed with a Varian CP-3800 gas chromatograph equipped with a DB-5 capillary column (30 m × 0.25 mm; coating thickness 0.25 μm) and a Varian Saturn 2000 ion trap mass detector. Analytical conditions: injector and transfer line temperatures 250 and 240 °C, respectively; oven temperature programmed from 60 to 240 °C at 3 °C/min; carrier gas helium at 1 mL/min.

### 4.3. UHPLC-UV-MS/MS Analysis

Aromatic waters were diluted in MeOH (1:1, *v*/*v*), centrifuged (4000 rpm) and injected (5 μL injection volume) into the LC system composed by a Vanquish Flex Binary UHPLC coupled with a Vanquish DAD and a Q Exactive Plus mass spectrometer, Orbitrap-based FT-MS system, equipped by an ESI source (Thermo Fischer Scientific Inc., Bremem, Germany). Elution was performed on a Kinetex^®^ Biphenyl column (100 × 2.1 mm, 2.6 μm) provided of a SecurityGuard^TM^ Ultra Cartridges (Phenomenex, Bologna, Italy), using formic acid in MeOH 0.1% *v*/*v* (solvent A) and formic acid in H_2_O 0.1% *v*/*v* (solvent B) as eluent and developing a linear solvent gradient of increasing 5 to 55% A within 15 min, at a flow rate 0.3 mL/min. During analysis, autosampler and column oven temperatures were maintained at 4 and 35 °C, respectively. UV data were registered using 254, 280 and 325 nm as preferential channels. A positive ion mode was used for ESI interface in a scan range of *m*/*z* 150–1200 and spectra were acquired both in full (70,000 resolution, 220 ms maximum injection time) and data dependent-MS/MS scan (17,500 resolution, 60 ms maximum injection time). The following ionization parameters were used: spray voltage 3500 V, capillary temperature 300 °C, sheath gas (N_2_) 20 arbitrary unit, auxiliary gas (N_2_) 3 arbitrary unit, collisionally activated dissociation (HCD) 18 eV. Data were elaborated with Xcalibur software.

### 4.4. NMR Analysis

NMR experiments were obtained on a Bruker Avance III 400 MHz spectrometer. All the samples were prepared by adding 60 μL of D_2_O as internal lock to 600 μL of each hydrolate. Pre-saturation was carried out with a relaxation delay (d1 = 2 s) and mixing time (d18 = 0.8 s). In both cases the number of scans was 64 and partial suppression of the solvent signals around 4.80 ppm was achieved.

### 4.5. Insect Rearing

*T. confusum* was reared at room temperature, 65% R.H., natural photoperiod, in PVC boxes (20 × 25 × 15 cm) containing maize and wheat grains and covered by a nylon net allowing air exchange. Homogeneous adults (1 day old) were obtained by removing adults from the box and the daily newly emerged insects were used for the bioassays [78].

### 4.6. Insect Repellence Bioassay

The bioassays were conducted following the method described by Bedini and colleagues [29] with some modifications. Preliminary tests were conducted to determine the appropriate range of concentration of the Lavandin hydrosols. Half filter paper disks (Whatman no. 1 filter paper, 10 cm Ø) was treated with 500 μL of different solutions containing 10, 50, 100, 120, 150 and 300 μL of Lavandin hydrosols to obtain hydrosol concentrations in the paper ranging from 0.4 to 12 μL·cm^−2^. As positive control, the synthetic repellent MR-08 (Menthol propyleneglycol carbonate) purchased from Parchem (USA) was tested in ethanolic solution at concentrations ranging from 0.00064 to 0.01274 μL·cm^−2^. The treated filter paper disks were then dried under a fan. After water evaporation, two half filter paper disks, one treated with the hydrosol solutions and the other treated with 500 μL of deionized water only (control) were put in the two halves of the bottom of a polystyrene Petri dish (10 cm Ø) to cover the entire surface. Twenty unsexed adult insects were then introduced in each Petri dish, and the lid was sealed with self-sealing film (Parafilm). The Petri dishes were maintained at 25 ± 1 °C, 65% R.H., in the dark. The number of the insects present on the two halves of the Petri dish was recorded after 1, 3 and 24 h from the beginning of the test. Five replicates were performed for each assay, and the insects were used only once. The percent repellence (PR) of essential oil and of each volatile compound was calculated by the formula: PR (%) = [(Nc − Nt) / (Nc + Nt)] × 100 where Nc is the number of insects present in the control half paper and Nt the number of insects present in the treated one. Data were processed by one-way between-groups univariate analysis of covariance (ANCOVA) with the hydrosol as fixed factor. The hydrosols concentration was considered as covariate in the model and its effect was controlled in the analysis. Post hoc comparisons were performed using Bonferroni corrections for multiple comparisons. The estimated marginal (EM) means of the insect pests’ mortality are reported. RC_50_ values of the two hydrosols were calculated by Log-probit regressions and reported as toxicological endpoints for comparison. Statistics were performed by SPSS 22.0 software (IBM SPSS Statistics, Armonk, North Castle, NY, USA).

### 4.7. In Vitro Germination Assay

To evaluate the effect of flowers and stems aromatic waters on the germination of seeds, the common commercial seeds of *Raphanus sativus* were selected. The seeds of this species were placed in 15 cm diameter Petri dishes (50 seeds each) lined with filter paper (Whatman No. 1, Whatman, Maidstone, UK) that was moistened with 5 mL of distilled water (control) or with 5 mL of each aromatic water. The Petri dishes were then incubated at 25 °C in climatic chambers equipped with fluorescent tubes (THL PHI- LIPS 20W/33, Philips, Amsterdam, Holland) producing white light (about 100 mol m^−2^ s^−1^), using a 12 h/12 h photoperiod. The number of germinated seeds was evaluated every 2 days (radicle appearance) until no further emergence was observed. Final germination was scored after 10 days. The germination percentage (GP) was calculated using the following equation: GP = S/T × 100, where S is the number of seed germinated during the assay and T is the total number of seeds used for the test. Data were expressed as mean ± standard deviation of three experiments.

### 4.8. Stability Assay

Colony count was performed on flowers aromatic water in an external laboratory by the inoculation in a nutrient agar culture medium following the indication of UNI EN ISO 6222:2001. This enumeration of culturable microorganisms test allowed the counting of colonies at 36 and 22 °C. The experiment was repeated at time 0, at months 6 and 12. Moreover, at time 0, the total Coliform counts (AFNOR BRD 07/20-03/11), *Pseudomonas aeruginosa* test (UNI EN ISO 16266:2008) and *Clostridium perfringens* (including spores) test (Italian D.Lgs. 2 febbraio 2001, n. 31) were also performed.

## 5. Conclusions

This work aimed to evaluate, for the first time, flowers and stems Lavandin aromatic waters. Both extracts were analysed by GC-MS, which indicates semi-quantitative differences between the major metabolites including linalool, 1,8-cineole, camphor, linalyl acetate and 4-terpineol. ^1^H-NMR and LC-MS analyses confirmed the presence of the main volatile components determined by GC-MS analyses, while not volatile compounds such as polar phenols were not found in the aromatic waters. The repellence bioassay against *T. confusum* showed a good activity for both hydrosols extracts with RD_50_ values of 3.6 and 3.3 µL cm^−2^ for the flowers and stems, respectively; at the higher concentrations, however, the hydrosol extract from the flowers is expected to be more effective than the one from the stems. The effect of the flowers and stems aromatic water of Lavandin on seed germination of *Raphanus sativus* was also evaluated. Results showed that seed germination was completely inhibited by flowers hydrolate, having therefore a possible application as natural herbicide. Significant allelopathic effect on germination and radical length inhibition was also observed for essential oil of Lavandin [79]. The potential use of the aromatic water instead of the essential oil could represent a great advantage especially in term of request of raw material versus finished product’s yield as better explained below. Moreover, using a water-based extract rather than an oily one looks more applicable and compatible with the actual reality of working in the field in the agricultural context. Further toxicological assays especially on soil microflora, however, are needed prior any possible application of this extract in crop management.

Considering hydrosols as main products of aromatic plants manufactory implies a series of radical shifts that are in line with certain ecopharmacognosy principles. The case of Lavandin here presented indicates the possibility to use a small piece of land (3 hectares, of which only around 0.5 hectares are dedicated to Lavandin cultivation) to develop a complete manufactory chain, from plant installation to a finished product. In a piece of land of such a small dimension, the production of Lavandin essential oil would be, and actually was, really limited. Aromatic water production, instead, allow the manufacturing of a higher amount of finished product that can become straightaway part of the company portfolio. Even if only the flowers were selected for the distillation, the balance between the plant materials used versus spendable finished product is still positive for the aromatic water compared with the essential oil manufactory. In fact, on the basis of the data acquired in this work (see Section 4.1 in Material and Methods), for 1 kg of plant material (including both flower and stems), it is estimated a yield of around 30 mL of essential oil, and around 830 mL of the improved aromatic water (obtained only from the flowers); considering that the essential oils are usually sold in the market in bottles of 10 mL, while aromatic waters in bottles of 100 mL, the number of finished product pieces is more than the double for the latters compared to the firsts. Moreover, following the proposed manufactory strategy, the stems of Lavandin remain virgin, not yet processed, plant material that could be used to develop other product lines including, for instance, those related with the textile manufactory; in this case, however, further researches are necessary to better define this potential.

By developing new product lines using fewer amounts of plant material and low-tech agricultural approach as for the Lavandin case here presented, the present research wants to push toward the implementation of small-scale agro-businesses; a kind of action that can be considered one of the relevant solutions to address climate change and human health issues, therefore in line with the general scopes of ecopharmacognosy.

## Figures and Tables

**Table 1:** Chemical composition (%) of Lavandin flowers and stem aromatic waters by HeadSpace, Solid Phase Micro Extraction coupled with Gas Chromatography Mass Spectrometry (HS-SPME-GC/MS).

**N.**	***t*_R_**	**LRI**	**Component**	**Relative Content %**	
				**Flowers**	**Stems**
1	3.50	867	1-hexanol	0.2	tr
2	6.05	986	6-methyl-5-hepten-2-one	-	0.1
3	6.18	992	myrcene	1.7	1.4
4	6.82	1013	*n*-hexyl acetate	0.2	tr
5	7.40	1034	1,8-cineole	25.4	28.9
6	7.97	1050	(*E*)-β-ocimene	0.3	0.6
7	8.85	1075	*cis*-linalool oxide (furanoid)	0.1	tr
8	9.43	1088	terpinolene	0.2	0.5
9	9.92	1099	linalool	43.8	34.4
10	11.00	1130	allo ocimene	tr	0.3
11	11.54	1144	camphor	12.8	15.4
12	12.53	1166	borneol	4.3	4.0
13	12.96	1178	4-terpineol	4.5	2.7
14	13.57	1190	α-terpineol	1.8	2.2
15	15.01	1233	isobornyl formate	0.1	0.3
16	15.45	1243	hexylisovalerate	0.3	0.5
17	15.67	1245	cumin aldehyde	0.2	0.4
18	16.27	1258	linalyl acetate	2.1	0.4
19	16.45	1260	geraniol	-	0.2
20	17.52	1285	isobornyl acetate	tr	0.2
21	17.82	1289	lavandulyl acetate	0.5	1.0
22	19.57	1331	hexyl tiglate	tr	0.2
23	21.02	1365	neryl acetate	0.2	0.4
24	21.86	1383	geranyl acetate	0.3	1.1
25	22.03	1391	7-episesquitujene	tr	0.2
26	23.22	1418	β-caryophyllene	0.2	1.3
27	24.91	1458	(*E*)-β-farnesene	0.2	1.4
28	25.84	1480	germacrene D	tr	0.5
29	27.13	1514	geranyl isobutyrate	tr	0.6
30	29.98	1581	caryophyllene oxide	tr	0.2
31	34.01	1683	α-bisabolol	-	0.1
Total				99.4	99.5
**Class of Compounds**				**Relative Content %**	
				**Flowers**	**Stems**
Monoterpene Hydrocarbons				2.2	2.5
Oxygenated Monoterpenes				96.1	92.5
Sesquiterpene Hydrocarbons				0.4	3.4
Oxygenated Sesquiterpenes				tr	0.3
Non Terpene Derivatives				0.7	0.8

LRI = linear retention index; tr = traces.

**Table 2:** Adjusted estimated marginal (EM) means of the repellence of Lavandin flowers and stems aromatic waters and of the synthetic repellent MR-08 against *Tribolium confusum* adults.

Extracts	Mean ^1^ ± SE	95% Confidence Interval	
		Lower Bound	Upper Bound
Flowers	49.82 ± 5.02	50.33	74.34
Stems	47.82 ± 5.02	48.00	72.00
MR-08	73.85 ± 7.67	58.57	89.14

^1^ Data are expressed as repellence % ± standard error (SE). Covariate (concentration) was evaluated at 3.84 μL·cm^−2^.

**Table 3:** Median repellent dose (RD_50_) of Lavandin flowers and stems aromatic waters and of the synthetic repellent MR-08 against *Tribolium confusum* adults.

Extracts	RD_50_^1^	RD_99_^2^	Slope ^3^	Intercept	*χ*2 (df)	*P*
**Flowers**	3.584(1.596–6.076)^4^	29.300(12.222–2543.698)	2.55 ± 0.30	−1.41 ± 0.20	18.20 (4)	0.001
**Stems**	3.262(1.814–5.420)^4^	136.069(38.175–5705.529)	1.45 ± 0.19	−7.37 ± 0.13	7.29 (4)	0.121
**MR-08**	0.001(0.000–0.003)	0.187(0.038–57.276)	1.08 ± 0.32	2.68 ± 0.69	0.78 (2)	0.678

^1^ Concentration of the extract that repels 50% of the exposed insect; ^2^ concentration of the extract that repels 99% of the exposed insect. Data are expressed as μL·cm^−2^; in brackets, the confidence interval; ^3^ Probit model: PROBIT(p) = Intercept + BX (Covariates X are transformed using the base 10.0 logarithm); (df), degrees of freedom; *P*, Pearson Goodness-of-Fit Test. ^4^ since *p* < 0.150, a heterogeneity factor is used in the calculation of confidence limits.

**Table 4:** Inhibition of *Raphanus sativus* seed germination by Lavandin flowers and stems aromatic waters.

	Number of Germinated Seeds ^1^	GP(%)^2^
Control	44 ± 0.3	88
Aromatic Water Flowers	0 ± 0.0	0
Aromatic Water Stems	12 ± 0.5	24

^1^ The number of germinated seeds was scored after 10 days; ^2^ GP = percentage of germination.
